# Compound heterozygosity of a novel missense variant and exonic deletion in hypomyelinating leukodystrophy 15

**DOI:** 10.1007/s10048-026-00885-4

**Published:** 2026-02-21

**Authors:** Akihiko Mitsutake, Takashi Matsukawa, Kenta Orimo, Kunihiro Ueda, Tomonari Seki, Yasushi Shiio, Jun Mitsui, Hiroyuki Ishiura, Harushi Mori, Shoji Tsuji, Tatsushi Toda

**Affiliations:** 1 Department of Neurology, Graduate School of Medicine, The University of Tokyo, Tokyo, Japan; 2 Department of Neurology, Tokyo Teishin Hospital, Tokyo, Japan; 3 Department of Precision Medicine Neurology, Graduate School of Medicine, The University of Tokyo, Tokyo, Japan; 4 Department of Neurology, Dentistry, and Pharmaceutical Sciences, Okayama University Graduate School of Medicine, Okayama, Japan; 5 Department of Radiology, School of Medicine, Jichi Medical University, Tochigi, Japan; 6 Institute of Medical Genomics, International University of Health and Welfare, Chiba, Japan; 7 National Center Hospital, National Center of Neurology and Psychiatry, Tokyo, Japan; 8 Department of Neurology, Graduate School of Medicine, The University of Tokyo, 7-3-1 Hongo, Bunkyo-ku, Tokyo, 113-8655 Japan

**Keywords:** Hypomyelinating leukodystrophy, EPRS1, Structural variant, Exon deletion, Nonsense‑mediated decay, Whole‑genome sequencing

## Abstract

Hypomyelinating leukodystrophy 15 (HLD15) results from biallelic pathogenic variants in *EPRS1*, but exonic deletions have not been reported. We describe a 40-year-old woman with mild intellectual disability, ataxia, dystonia, and MRI showing hypomyelination. Whole-exome sequencing identified a heterozygous missense variant in the prolyl-tRNA synthetase domain of *EPRS1* (c.3430 C > G; p.Leu1144Val, NM_004446.3), without second variant. Whole-genome sequencing revealed a heterozygous 220-bp deletion spanning exon 15 (c.1743-30_1932del), and segregation analysis confirmed compound heterozygosity. RT-PCR from lymphoblastoid cells demonstrated exon-15 skipping leading to a frameshift (p.Asn582Serfs*10) and nonsense-mediated decay, leaving predominant expression of the paternally inherited missense allele. These findings support loss-of-function for the deletion and classify c.3430 C > G as likely pathogenic under ACMG/AMP criteria (PM1, PM2, PM3, PP3). This case represents the first exonic deletion reported in *EPRS1*. The relatively mild, adult-onset phenotype broadens both mutational and clinical spectra of HLD15 and highlights the importance of structural-variant analysis when only a single candidate variant is detected in recessive leukodystrophy.

**Supplementary Information:**

The online version contains supplementary material available at 10.1007/s10048-026-00885-4.

## Introduction

Hypomyelinating leukodystrophies (HLDs) are a heterogeneous group of genetic neurological disorders characterized by insufficient myelin formation in the brain. Clinical features typically include motor and cognitive impairment with onset in early childhood or adolescence [1]. Recent advances in next-generation sequencing have improved the genetic diagnosis of HLDs, identifying a variety of genes essential for myelin production and maintenance as causative genes. Among these, the involvement of genes encoding cytoplasmic aminoacyl-tRNA synthetases (ARSs) has recently been increasingly recognized. In particular, biallelic pathogenic variants in *AARS1*, [2] *DARS1*, [3] *EPRS1*, [4] and *RARS1* [5]have been known to cause HLDs. *EPRS1*, one of the ARS genes, encodes the bifunctional enzyme glutamyl-prolyl-tRNA synthetase and has been identified as a causative gene for HLD15, an autosomal recessive disorder [4, 6]. 

In this study, we report a case of HLD15 due to compound heterozygous pathogenic variants in *EPRS1*, with a novel missense variant and an exonic deletion. The present case was characterized by mild clinical phenotype.

## Case description

The patient was born to healthy parents without a family history of neurological disorders nor consanguinity. From approximately 6–7 years of age, her academic performance was slightly below average, and she had mild coordination difficulties in sports. However, these features were subtle and nonspecific, and their relevance to the later neurological disorder remains uncertain. After graduating high school, she began working in an office job.

**Fig. 1:**
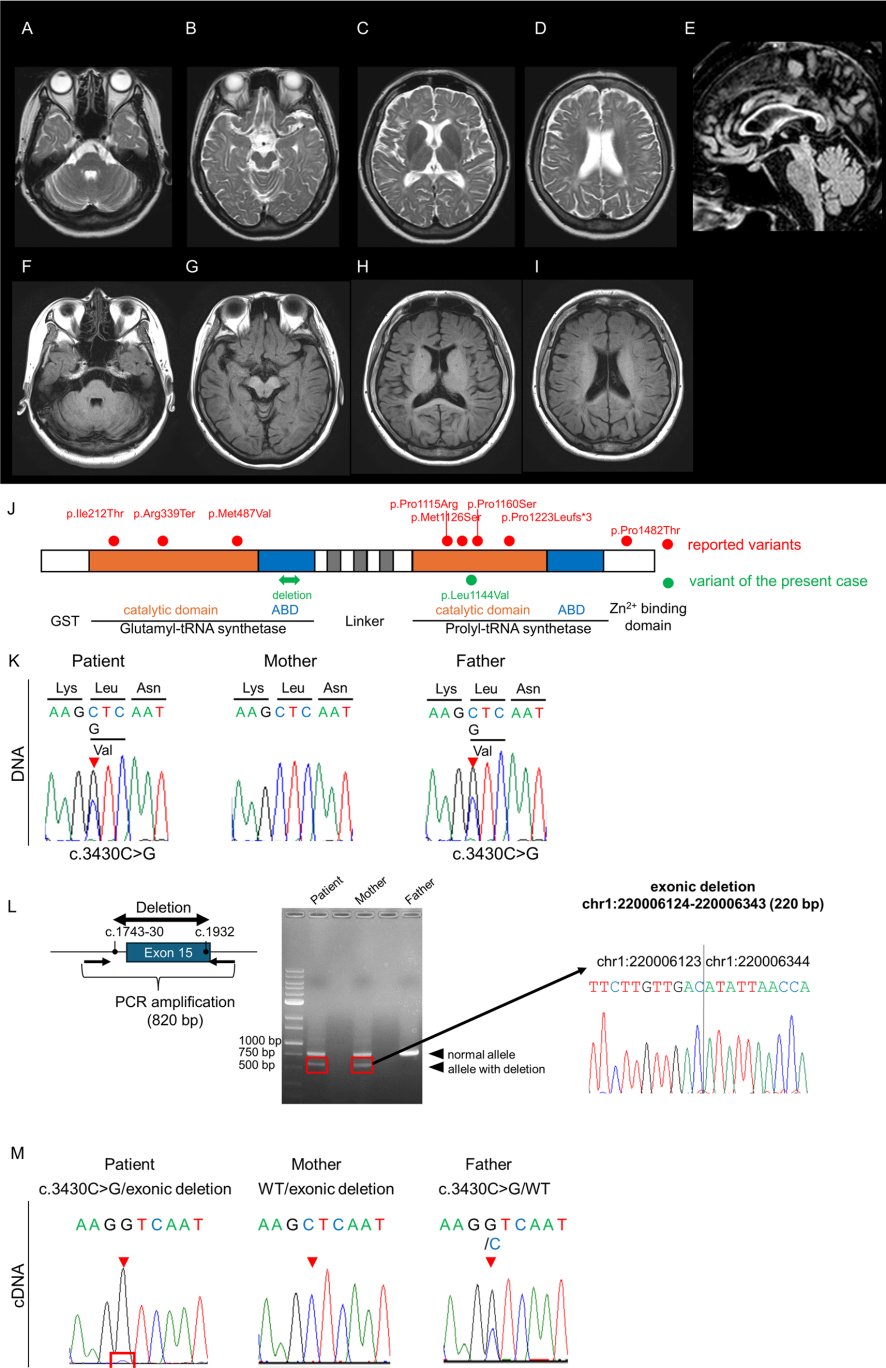
Brain MRI and genetic analysis of the present case. (**A–D**) Axial T2-weighted images obtained at age 40 show hyperintensity in the subcortical white matter. No abnormal signal intensity is observed in the middle cerebellar peduncle. (**E**) Sagittal T1-weighted image demonstrates thinning of the corpus callosum. (**F–I**) Axial T1-weighted images show slight hyperintensity of the periventricular white matter and basal ganglia relative to cortex. (**J**) Schematic representation of the EPRS1 gene indicating the positions of pathogenic variants within functional domains. ABD, anticodon-binding domain. (**K**) Sanger sequencing of EPRS1 reveals a heterozygous missense variant, c.3430 C > G (p.Leu1144Val), in the patient and her father. (**L**) Agarose gel electrophoresis of PCR products from genomic DNA using primer pairs flanking the deleted region shows that the patient and her mother share the deletion (left). The electropherogram of the breakpoint junctions indicates a 220-bp deleted region (right). (**M**) Electropherogram of RT-PCR products demonstrates monoallelic expression of the c.3430 C > G variant in the patient. The mother does not carry the variant, while the father is heterozygous for c.3430 C > G

At the age of 32, she began experiencing difficulty with writing, accompanied by neck tremors. Her writing difficulties gradually worsened, and she later developed dysarthria and incoordination. By age 40, the patient showed worsening gait instability and tremor, which prompted her to visit an outpatient clinic. Brain MRI revealed extensive cerebral white matter atrophy accompanied by hyperintensity on T2-weighted images, with thinning of the corpus callosum (Fig. 1A–E). T1-weighted images showed slight hyperintensity of the periventricular white matter and basal ganglia relative to cortex (Fig. 1F–I). Leukoencephalopathy with unknown etiology was suspected, and she was referred to our hospital for further evaluation.

Neurological examination revealed slurred speech, ataxia in the limbs and trunk, focal hand dystonia, and postural tremor in the neck. In cognitive assessments, the patient scored 26 out of 30 on the Mini-Mental State Examination, 23 of 30 on the Japanese version of the Montreal Cognitive Assessment, and 12 of 18 on the Frontal Assessment Battery, indicating mild cognitive impairment. HLD was suspected, and genetic analysis was performed.

The patient and her parents provided written informed consent. The study was approved by the institutional review board. Genomic DNA samples were prepared from peripheral blood leukocytes obtained from the patient and her parents.

We conducted Whole-exome sequencing (WES) of the patient using the SureSelect Human All Exon V6 + UTRs kit (Agilent Technology, Santa Clara, CA) with the HiSeq 2500 platform (Illumina, San Diego, CA). The sequences were aligned to the GRCh37/hg19 reference genome using the Burrows Wheeler Aligner, and variants were called with SAMtools [7, 8]. After initial variant filtering (quality score > 20 and minor allele frequency < 0.01) against our in-house exome database of 1,163 Japanese controls, 491 rare variants remained. We then prioritized variants in genes associated with hypomyelinating leukodystrophies and related neurodevelopmental disorders (Supplementary Table 1). This analysis yielded a single plausible candidate consistent with the phenotype: a heterozygous missense variant in *EPRS1* (c.3430 C > G; p.Leu1144Val) (NM_004446.3). Trio-based analysis did not identify any plausible *de novo* variants consistent with the phenotype relevant to leukodystrophy.

This variant has not been registered in HGMD, ClinVar, gnomAD v4.1.0 (allele frequency = 0), or ToMMo 54KJPN and affects an amino acid conserved across vertebrates. It is predicted to be deleterious, with a Combined Annotation Dependent Depletion v1.6 Phred score of 26.6. The variant is located in the Prolyl-tRNA synthetase (ProRS) domain of *EPRS1*, a region where most pathogenic variants have been reported, including the pathogenic c.3377T > C (p.Met1126Thr) variant in the same exon (Fig. 1J). Segregation analysis confirmed that the missense variant was paternally inherited (Fig. 1K). However, no other SNVs or short indels were detected in *EPRS1*.

Whole-genome sequencing (WGS) was performed at the National Center for Global Health and Medicine using the NovaSeq 6000 platform (Illumina, San Diego, CA) with 150-bp paired-end reads at a target depth of 30× [9]. Reads were aligned to the GRCh38/hg38 reference genome, and variant calling was conducted using Parabricks v3.1.0 (NVIDIA, Santa Clara, CA), which accelerates GATK-recommended analyses through GPU processing [10]. 

Structural variant (SV) analysis was performed on WGS BAM files aligned to GRCh38. SVs were identified with the Manta structural variant caller [11]. Manta called a 220‑bp deletion in *EPRS1* involving part of exon 15 [NC_000001.11:g.220006124_220006343del; NC_000001.11(NM_004446.3):c.1743-30_1932del]. To define the exact breakpoints, we designed PCR primers flanking the breakpoint junction. Direct sequencing of the PCR products confirmed the deletion [NC_000001.11:g.220006124_220006343del; NC_000001.11(NM_004446.3):c.1743-30_1932del], concordant with the Manta call. This SV was not registered in gnomAD SVs v4.1.0 (allele frequency = 0). The exon 15 deletion was maternally inherited (Fig. 1L). The paternally inherited missense variant and the maternally inherited exon 15 deletion were present *in trans*, establishing compound heterozygosity. When exome sequence data were reanalyzed, read depths derived from exon 15 were confirmed to be decreased to approximately 0.5 (Supplementary Fig. 1).

To assess the splicing consequence of c.1743-30_1932del, we performed RT-PCR using junction-spanning primers (Supplementary Table 2) on cDNA synthesized from total RNA of lymphoblastoid cells from the proband and her parents. RT-PCR revealed a predominant full-length product, but direct sequencing showed a minor transcript consistent with exon 15 skipping. To examine whether transcripts from the deletion allele undergo nonsense-mediated mRNA decay (NMD), we used the paternally inherited c.3430 C > G variant in exon 24 as an allele-specific marker and performed RT-PCR across exons 22–26. In the proband, sequencing of the RT-PCR product showed predominant expression of the G allele with only a faint residual C peak, whereas the father remained heterozygous and the mother lacked the variant (Fig. 1M). These results indicate that exon 15 skipping from the maternally inherited deletion allele leads to NMD, resulting in relative overrepresentation of the paternal allele.

c.1743-30_1932del was thus considered a loss-of-function variant, and was classified as likely pathogenic according to ACMG guidelines, meeting criteria PVS1 and PM2 [12]. In light of this, the missense variant c.3430 C > G was classified as likely pathogenic according to ACMG/AMP guidelines, meeting criteria PM1, PM2, PM3, and PP3 [12]. 

## Discussion

*EPRS1* encodes a bifunctional aminoacyl-tRNA synthetase, with an N-terminal glutamyl-tRNA synthetase (GluRS) domain and a C-terminal ProRS domain joined by a flexible linker [14]. The enzymatic functions of GluRS and ProRS are integrated within a single polypeptide [15]. Patients with biallelic pathogenic variants in *EPRS1* typically present with motor and cognitive impairment, as summarized in Table 1 [4, 6, 13]. Mendes et al. described four individuals carrying primarily missense changes in the ProRS domain; all showed motor regression, ataxia, spasticity and dystonia. Brain MRI showed HLD with thinning of the corpus callosum, and two patients showed T2 hyperintensities in the middle cerebellar peduncles [4]. Jin et al. identified a four‑year‑old girl presenting with psychomotor developmental delay, seizures, and deafness. The patient harbored compound heterozygous missense variants in the GluRS domain. Despite profound enzymatic defects in vitro, her MRI did not show hypomyelination [13]. More recently, Khan et al. identified two siblings homozygous for a Zn^2+^‑binding–domain variant; these patients exhibited severe neurodevelopmental delay, ataxia, progressive spasticity and microcephaly, attributable to disrupted m^6^A modification of EPRS1 mRNA and consequent protein depletion [6]. Taken together, these studies suggest that the affected domain may influence the clinical features. ProRS-domain variants often show the typical HLD15 imaging pattern, whereas GluRS-domain variants may be associated with seizures, deafness, and less obvious hypomyelination, though the number of reported cases is limited.

Notably, the second pathogenic allele was not detected by the initial short-read WES analysis. Although short-read WES is optimized for the detection of SNVs and small indels, it has limited sensitivity for single-exon structural variants. Therefore, WGS was performed when only a single heterozygous pathogenic variant was identified. Subsequent reanalysis of the WES data revealed reduced read depth over exon 15, indicating that the deletion might have been suspected at the exome level. However, single-exon copy-number changes inferred from read-depth analysis alone are often inconclusive and require independent confirmation.

Compared with previous reports, the present patient showed a milder clinical phenotype. The identified missense variant is located in the ProRS-domain, consistent with prior reports of pathogenicity in this region. Additionally, the exon 15 deletion lies in the GluRS region. Our RT‑PCR findings demonstrated exon-15 skipping, leading to NMD of the maternal allele. Prior functional studies of ProRS‑domain EPRS1 variants showed reduced ProRS aminoacylation/tRNA‑charging activity and were associated with early‑onset, progressive HLD15. Given that the deletion allele is functionally silenced by NMD, the residual EPRS1 function in the present case is expected to be determined by the p.Leu1144Val allele. The mild, adult‑onset phenotype is therefore consistent with relatively preserved residual activity compared with previously reported ProRS‑domain cases, although direct enzymatic assays are needed for confirmation. To our knowledge, this is the first report of exonic deletion in *EPRS1*, and the relatively mild presentation further broadens the known genotype–phenotype spectrum of HLD15.

**Table 1:** Clinical and radiological features of patients with bi-allelic *EPRS1* variants

No.	Age at onset	Variant (Allele 1)	Variant (Allele 2)	Key Clinical Features	MRI Findings
1^4^	3 years	c.3344 C > G (p.Pro1115Arg)	(homozygous)	cognitive regression; ataxia; spasticity; dystonia; intention tremor	hypomyelination; supratentorial atrophy; corpus callosum thinning
2^4^	14 years	c.1015 C > T (p.Arg339Ter)	c.3344 C > G (p.Pro1115Arg)	cognitive and motor regression; ataxia; spasticity; dystonia; intention tremor; epilepsy	hypomyelination; supratentorial atrophy; corpus callosum thinning; T2 hyperintensity (cerebellar white matter)
3^4^	infancy	c.3478 C > T (p.Pro1160Ser)	c.3667delA (p.Thr1223Leufs3*)	motor regression; ataxia; dystonia; pyramidal signs; failure to thrive; dysphagia	hypomyelination; supratentorial atrophy; cerebellar atrophy; corpus callosum thinning; T2 hyperintensity (pyramidal tract, cerebellar white matter, middle cerebellar peduncle)
4^4^	1 year	c.3377T > C (p.Met1126Thr)	(homozygous)	motor regression; ataxia; dystonia; athetoid movements; pyramidal signs; dysphagia	hypomyelination; supratentorial atrophy; cerebellar atrophy; corpus callosum thinning; T2 hyperintensity (middle cerebellar peduncle)
5^13^	9 months	c.1459 A > G (p.Met487Val)	c.635 T > C (p.Ile212Thr)	developmental delay; epilepsy	T2 hyperintensity (left lateral periventricular white matter); cortical atrophy
6^6^	infancy	c.4444 C > A (p.Pro1482Thr)	(homozygous)	developmental delay; intellectual disability; ataxia; spasticity; hypotonia; failure to thrive	hypomyelination; supratentorial atrophy; cerebellar atrophy; corpus callosum thinning
7^6^	infancy	c.4444 C > A (p.Pro1482Thr)	(homozygous)	developmental delay; intellectual disability; ataxia; hypotonia; spasticity; hypotonia	hypomyelination; supratentorial atrophy; cerebellar atrophy; corpus callosum thinning
8*	32 years	c.3430 C > G (p.Leu1144Val)	c.1743-30_1932del	intellectual disability; ataxia; dystonia	hypomyelination; cerebellar atrophy; corpus callosum thinning

*The present case

## Supplementary Information

Below is the link to the electronic supplementary material.

Supplementary Material 1

## Data Availability

No datasets were generated or analysed during the current study.
