# Supplementary material for: Compound heterozygosity of a novel missense variant and exonic deletion in hypomyelinating leukodystrophy 15
Source: Neurogenetics. 2026 Feb 21;27(1):16. doi: 10.1007/s10048-026-00885-4 (PMC12923470; doi:10.1007/s10048-026-00885-4)
Supplement: Supplementary file 1 — Supplementary Material 1 [file 10048_2026_885_MOESM1_ESM.docx]

**Supplementary Materials**

**Compound Heterozygosity of a Novel Missense and Copy Number Variant in Hypomyelinating Leukodystrophy 15**

**Supplementary Table 1. Genes related to leukodystrophy**

*ADGRV1, ADSL, ALDH7A1, ALG13, ARG1, ARHGEF9, ARX, ASAH1, ATP1A2, ATP1A3, ATP6AP2, ATP7A, BRAF, CACNA1A, CACNB4, CDKL5, CHD2, CHRNA2, CHRNA4, CLCN4, CLN3, CLN5, CLN6, CLN8, CNTNAP2, COX6B1, CSTB, CTSD, CYB5R3, DEPDC5, DNAJC6, DNM1, DOCK7, EEF1A2, EFHC1, FLNA, FOLR1, FOXG1, GABBR2, GABRA1, GABRB2, GABRB3, GABRD, GABRG2, GLB1, GLRA1, GPR56, GRIN1, GRIN2A, GRIN2B, HCN1, HDAC4, HEXA, HEXB, HNRNPH1, HNRNPU, IQSEC2, KCNB1, KCNH1, KCNMA1, KCNQ2, KCNQ3, KCNT1, LGI1, LIAS, MBD5, MECP2, MEF2C, MFSD8, MTHFR, MTOR, NDE1, NEDD4L, NRXN1, PAFAH1B1, PCDH19, PDHA1, PIGA, PIGV, PLCB1, PNKD, PNKP, PNPO, POLG, PPT1, PRRT2, RELN, SCN1A, SCN1B, SCN2A, SCN8A, SHANK3, SIK1, SLC1A3, SLC2A1, SLC9A6, SLC13A5, SLC19A3, SLC25A22, SLC35A2, SPTAN1, SRPX2, ST3GAL5, STRADA, STXBP1, SYNGAP1, SYNJ1, SZT2, TBC1D24, TCF4, TPP1, TSC1, TSC2, TUBA1A, UBE3A, WDR45, ZEB2*

Supplementary Table 2. Primers used for RT-PCR

1. To assess whether the c.1743-30_1932del variant affects splicing

F ACCCAAAGAATCCTGAGGTT

R AAAGGAGCAGAGGTCTCATT

1. To detect c.3430C>G

F CCAGAGGTTGCTTGGGTTAC

R TCTGCAAGACCTCTTCCGCT


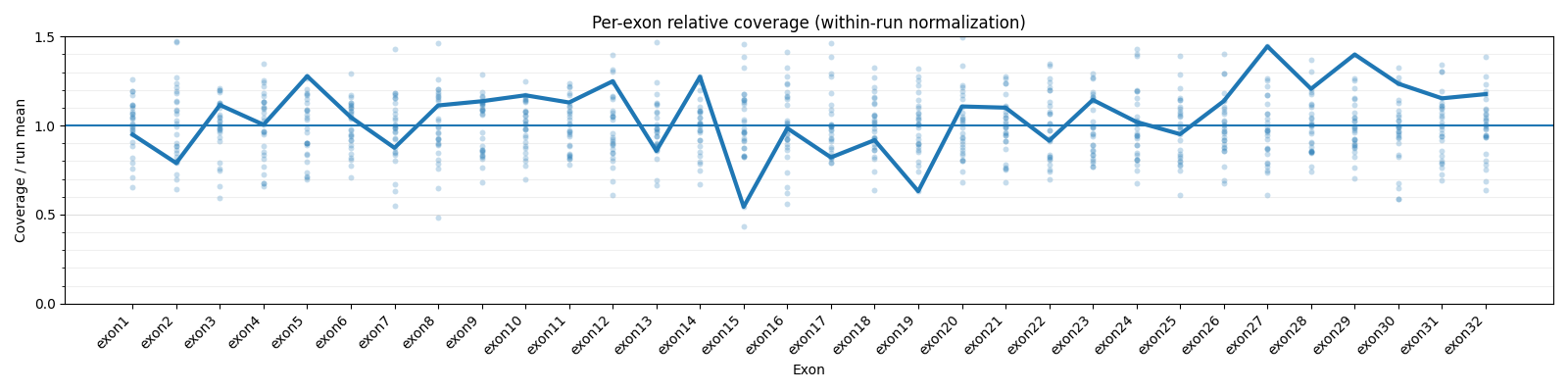


Supplementary Figure 1. Reanalysis of exon-level coverage of EPRS1 from whole-exome sequencing data

Exon-level read depth for *EPRS1* was calculated from whole-exome sequencing data and normalized to reads per million mapped reads (RPM). For each exon, the RPM-normalized coverage of the proband was further normalized to the mean coverage of other samples sequenced in the same run (within-run normalization). Individual dots represent exon-level relative coverage values from all samples in the same sequencing run, while the solid line indicates the proband. This analysis shows a marked reduction of relative coverage at exon 15 to approximately 0.5, whereas coverage across other exons remained near the expected diploid level.
